# Remimazolam-based precision anesthesia management combined with nasopharyngeal airway in gastroscopic surgery for a patient with obesity and difficult airway: a successful case report

**DOI:** 10.3389/fmed.2026.1762932

**Published:** 2026-02-11

**Authors:** Hui Ji, Mengran Xie, Hao Wang, Huanshuang Pei

**Affiliations:** Department of Anesthesiology, The Fourth Hospital of Hebei Medical University, Shijiazhuang, China

**Keywords:** difficult airway, gastroscopy, nasopharyngeal airway, obesity, obstructive sleep apnea hypopnea syndrome, remimazolam

## Abstract

**Background:**

Patients with moderate obesity, obstructive sleep apnea hypopnea syndrome (OSAHS), and an anticipated difficult airway are at high risk for hypoxemia and airway complications under deep sedation.

**Case:**

The initial procedural attempt was aborted due to propofol-induced respiratory complications, including pronounced coughing and severe hypoxemia, in a high-risk patient with obesity and a Mallampati Class III airway. The subsequent optimized anesthetic protocol involved proactively establishing a patent upper airway with a lubricated nasopharyngeal conduit. Sedation was then carefully induced using remimazolam, a novel ultra-short-acting benzodiazepine chosen for its superior cardiorespiratory stability. This strategy successfully maintained spontaneous ventilation and stable hemodynamics throughout the entire gastroscopy procedure, enabling immediate reversal with flumazenil and facilitating rapid postoperative recovery.

**Conclusion:**

This case demonstrates that for high-risk patients, the “precise sedation with preserved spontaneous breathing” strategy using remimazolam combined with a nasopharyngeal airway is a safe, effective, and reversible innovative anesthesia option.

Obesity has emerged as a global health concern, and patients with pathological obesity [body mass index (BMI) ≥35 kg/m^2^] frequently present with OSAHS, which poses significant challenges in anesthetic management (1). These individuals typically exhibit reduced residual lung function, increased oxygen consumption, and a heightened tendency for upper respiratory tract collapse (2). The risk of airway obstruction and severe hypoxemia is significantly increased under sedation or anesthesia. Moreover, obesity is an independent predictor of difficult airways, further increasing the risks associated with anesthesia (3).

Propofol is the most commonly utilized sedative in digestive endoscopic surgery; however, its pronounced respiratory and circulatory inhibitory effects are of particular concern. For patients with obesity having OSAHS and difficult airways, using propofol alone or as the main agent presents considerable risk (4). Therefore, it is crucial to explore safer and more controllable anesthetic regimens for this patient population.

Remimazolam is a novel ultra-short-acting benzodiazepine sedative that can be specifically antagonized by flumazenil. It has a rapid onset and elimination, mild respiratory and circulatory inhibition, and the characteristic of “conscious sedation,” providing an ideal choice for high-risk patients who require preservation of spontaneous respiration (5). In addition, the nasopharynge al airway (NPA),[media/image1.png] a simple and effective airway tool, can effectively maintain upper airway patency without the need for intubation, especially in sedation scenarios where spontaneous breathing is preserved (6).

This case report details the successful administration of gastroscopic anesthesia in a patient with moderate obesity, OSAHS, and an anticipated difficult airway, by combining remimazolam with NPA technology, and exploring its clinical innovation and application value.

## Case report

The patient is a 60-year-old male (height, 170 cm; weight, 100 kg; BMI, 34.6 kg/m^2^), classified as moderately obese. He was admitted due to abdominal distension persisting for over 2 years and the discovery of gastric polyps, identified 1 month prior. An endoscopic polypectomy was planned. The patient had a 30-year history of well-controlled hypertension and denied any history of diabetes or coronary heart disease. He also had a 30-year history of OSAHS and required nightly non-invasive assisted ventilation. Preoperative airway assessment revealed Mallampati grade III, indicating a potentially difficult airway. Other physical and laboratory examinations showed no significant abnormalities.

### First anesthesia procedure (October 22, 2024)

The initial anesthesia procedure was performed at an external hospital. At the time of the patient's entry into the operating room, vital signs were as follows: heart rate (HR) 72 beats/min, respiratory rate (RR) 17 breaths/min, blood pressure (BP) 168/86 mmHg, and pulse oxygen saturation (SpO_2_) 92% (without supplemental oxygen). A conventional intravenous anesthesia regimen was initiated with propofol. Immediately following administration, the patient exhibited a pronounced coughing response and rapidly developed severe hypoxemia (progressive decrease in SpO_2_), though the specific data and duration remain unknown. Jaw support and mask oxygenation were promptly applied, resulting in a gradual SpO_2_ increase. Due to this adverse event, the procedure was suspended. The patient regained consciousness and was returned to the ward, where he received symptomatic treatment, including spasmolysis and phlegm reduction. He recovered well after 1 week.

### Second anesthesia procedure (October 29, 2024, innovative plan)

After the patient entered the operating room, a peripheral intravenous access was established, and the patient was connected to the monitor. Vital signs were recorded as follows: HR 101 beats/min, RR 21 breaths/min, BP 162/85 mmHg, SpO_2_ 93% (without supplemental oxygen), bispectral index (BIS) 94. A meticulously designed anesthesia plan was implemented:

1. Preoperative preparation and airway management: the patient was positioned in the left lateral position. Oxygen was administered via a nasal cannula at 5 L/min. Mucosa surface anesthesia of the nasal cavity was achieved using 5 ml of Dakronin emulsion for 10 min, followed by 5 ml of 2% lidocaine for anesthesia of the right nasal cavity.

2. Bypass safe airway establishment: after 1 min, a 7.0# connector-type NPA, pre-lubricated with aubcaine gel, was inserted along the natural bending direction of the right nasal cavity. After confirming the correct position, connected the NPA directly to the anesthesia machine's breathing circuit. At this point, the patient's spontaneous respiratory rate was monitored at 18 breaths per minute, with a tidal volume of 450–550 ml.

3. Mucosa surface anesthesia of the nasopharyngeal and deep sedation enhancement: an additional 5 ml of 2% lidocaine was administered via the laryngeal anesthesia tube to anesthetize the pharyngeal cavity and minimize stimulation during gastroscopy. After 3 min, 25 mg (loading dose) of remimazolam (Jiangsu Hengrui Medicine Co., Ltd) was slowly administered intravenously. The BIS value decreased to 56. The gastroscope was successfully inserted without patient movement or coughing. The therapeutic procedure commenced.

4. Intraoperative management: the gastroscopy procedure lasted 13 min, during which the patient remained motionless. The patient maintained stable spontaneous breathing throughout the procedure, with a respiratory rate of 15~18 breaths/min, tidal volume of 250~400 ml, and excellent SpO_2_ at 99~100%. Hemodynamic parameters also remained stable: BP 130~140/90~98 mmHg; HR 60~90 beats/min. Additional remimazolam was administered to maintain BIS levels between 50 and 60. A total of 40 mg of remimazolam was used by the end of the procedure, with 100 ml of sodium acetate Ringer's solution infused.

Recovery and outcome: at the end of the surgery, 0.5 mg of Flumazenil was administered intravenously for antagonism. One minute later, the BIS rose to 88. The patient regained consciousness and reported having slept well, with no memory of the treatment process, and was very satisfied with this painless gastroscopic treatment. After a 30-min observation period in the Post-Anesthesia Care Unit (PACU), the patient was transferred back to the ward with an Modified Aldrete score >9 and no occurrence of nausea or vomiting. Twenty-four hours later, the patient did not complain of chest tightness, shortness of breath, dizziness, nausea, or other issues. He recovered and was discharged from the hospital 3 days after surgery without respiratory complications. Two months after surgery, the patient did not complain of discomfort at a telephone follow-up (Table 1).

**Table 1:** Case report timeline.

Item		**Timeline**
Preoperative	1	Gastric polyps for 1 month were discovered.
	2	The patient had a 30-year history of well-controlled hypertension and denied any history of diabetes or coronary heart disease. He also had a 30-year history of OSAHS and required nightly non-invasive assisted ventilation.
	3	Preoperative airway assessment revealed Mallampati grade III, indicating a potentially difficult airway.
	4	The patient required a painless endoscopic polypectomy.
Perioperative	5	The patient was admitted to the room, and BP, HR, SpO_2_, RR, and BIS were routinely monitored.
	6	The patient was positioned in the left lateral position and was connected to a nasal cannula for oxygen intake of 5 L/min.
	7	Mucosa surface anesthesia of the nasal cavity was achieved using 5 ml of Dakronin emulsion for 10 min, followed by 5 ml of 2% lidocaine for anesthesia of the right nasal cavity.
	8	After 1 min, a 7.0# connector-type NPA, pre-lubricated with aubcaine gel, was inserted along the natural bending direction of the right nasal cavity.
	9	After confirming the correct position, connected the NPA directly to the anesthesia machine's breathing circuit. At this point, the patient's spontaneous respiratory rate was monitored at 18 breaths per minute, with a tidal volume of 450–550 ml.
	10	An additional 5 ml of 2% lidocaine was administered via the laryngeal anesthesia tube to anesthetize the pharyngeal cavity and minimize stimulation during gastroscopy.
	11	After 3 min, 25 mg (loading dose) of remimazolam was slowly administered intravenously.
	12	BIS value decreased to 56. The gastroscope was successfully inserted without patient movement or coughing.
	13	The patient exhibited no body movements or coughing during endoscopic treatment, with stable vital signs. Remimazolam was administered as needed based on changes in the BIS, totaling 40 mg by the end of the procedure.
Postoperative	14	At the end of the surgery, 0.5 mg of Flumazenil was administered intravenously for antagonism.
	15	The patient regained consciousness and reported having slept well, with no memory of the treatment process, and was very satisfied with this painless gastroscopic treatment.
	16	After a 30-min observation period in the PACU, the patient was transferred back to the ward with an Modified Aldrete score >9 and no occurrence of nausea or vomiting.
	17	The patient was discharged 3 days after treatment.
	18	Two months after treatment, the patient did not complain of discomfort on telephone follow-up.

ECG, electrocardiography; SpO_2_, pulse oximetry; BIS, bispectral index; CT, computed tomography; BP, blood pressure; HR, heart rate; RR, respiratory rate; NPA, nasopharyngeal airway; PACU, post-anesthesia care unit.

## Discussion

Anesthesia management in the present case was particularly challenging due to the patient's moderate obesity, long-term severe OSAHS, Mallampati III airway, and a prior failed propofol-based anesthesia. The initial anesthesia failure revealed the limitations of traditional propofol regimens in high-risk patients, particularly due to their strong respiratory depressant effects and propensity to induce cough, which can easily trigger severe hypoxemia (4). The success of the second anesthesia procedure can be attributed to implementing an innovative strategy centered on: pre-established bypass airway, spontaneous breathing preservation, precise sedation, and reversible antagonism.

A pivotal element of this approach was the pre-placement of the NPA during the patient's conscious and cooperative state before sedation. This was not intended for routine ventilation, but served as a “preventive” safety measure to establish a “bypass safe airway”, circumventing the potentially collapsed base of the tongue and soft palate. It effectively prevented upper airway obstruction after sedation, ensuring smooth gas exchange throughout the procedure. Connecting anesthesia circuits to monitor respiratory parameters provided an objective basis for regulating sedation depth.

The precise sedative advantage of remimazolam was critical in the sedation regimen, offering pharmacokinetic characteristics ideally suited for this clinical scenario. Compared to propofol, remimazolam exerts a milder inhibition of respiratory drive, facilitating stable spontaneous breathing throughout the procedure, as confirmed by the stable SpO_2_, RR, and blood circulation data (5, 7). The patient's intraoperative BP and HR were stable, avoiding the common dose-related hypotension risk of propofol, which is particularly important for patients with hypertension (8). Additionally, predictability and reversibility were an advantage, as remimazolam is metabolized independently of liver and kidney functions, with minimal individual differences and short duration of action. Notably, its effects can be reversed with flumazenil (9), as demonstrated by the patient's prompt recovery within 1 min. The rapid reversal prevents the risk of secondary respiratory depression caused by residual sedative effects after surgery, which is crucial for patients with OSAHS.

Mucosa surface anesthesia of the nasopharyngeal is essential for minimizing irritation caused by nasopharyngeal tube insertion and gastroscopy procedures. This enabled completion of the surgery at a shallower depth of sedation, further reducing the dosage of sedatives and their side effects (10). The practice in this case further confirms the effectiveness of adequate topical anesthesia. In fact, remifentanil was prepared on standby and planned to be administered intravenously in case of body movement during gastroscope insertion. However, due to adequate topical anesthesia of the nasopharyngeal mucosa prior to the procedure, no body movement or coughing occurred during gastroscope insertion or manipulation, and thus remifentanil was not required. This result highlights the critical role of comprehensive topical anesthesia in avoiding noxious stimulation and reducing the need for additional sedative or analgesic agents.

Compared to the initial anesthesia procedure, the patient maintained significantly better oxygenation during the second anesthesia session. This improvement was not attributable to a single factor but rather resulted from the synergistic effects of pre-oxygenation, pre-placement of the NPA, and the milder respiratory depressant properties of remimazolam. Together, these elements constitute a comprehensive strategy of “prevention, safeguard, and optimization.” Specifically, pre-oxygenation provided a foundational oxygen reserve and extended the safe buffer time. The insertion of the NPA served as the critical structural safeguard, ensuring that airflow could pass smoothly through the tube even after sedation-induced muscle relaxation, thereby fundamentally preventing upper airway obstruction. The use of remimazolam represented an optimized pharmacological choice, characterized by weaker respiratory depression and the availability of a specific antagonist, allowing its sedative effects to be rapidly reversed. These combined factors cannot be equated with the use of propofol alone. This approach is not merely a drug substitution but rather a paradigm shift in anesthetic philosophy and management protocols, shifting the focus from sedation depth alone to maintaining airway patency and spontaneous breathing as the core of perioperative safety. For this high-risk patient, the safety and controllability of this strategy were far superior to the conventional approach relying solely on propofol.[media/image1.png]

The success of this case demonstrates the efficacy of the “dual insurance” technique, which combines remimazolam with NPA. The NPA physically ensures airway patency, while remimazolam offers pharmacological control, minimizing respiratory inhibition. This approach provides a novel and safer alternative for endoscopic surgery in patients with OSAHS, obesity, and difficult airways, avoiding the trauma and complexity of endotracheal intubation and the risk of deep sedation without airway protection.

For high-risk patients with comorbid obesity, OSAHS, and anticipated difficult airways, conventional anesthesia induction and sedation regimens pose substantial risks. This case report innovatively adopts a comprehensive management strategy based on remimazolam sedation combined with pre-implantation of NPA to ensure upper respiratory tract patency. This strategy leverages the pharmacological advantages of remimazolam, including mild respiratory inhibition, stable circulation, and rapid reversibility, alongside the mechanical advantages of the NPA tool, successfully achieving surgery while preserving spontaneous breathing and ensuring perioperative safety. It should be noted that the anesthetic technique of using remimazolam alone with surface anesthesia for nasopharyngeal airway placement is not suitable for all patients. This surgical approach is not applicable to every patient, which represents a limitation of this study. In conclusion, this study provides valuable clinical insights and a successful model for ensuring anesthesia safety in high-risk populations, warranting further evaluation and potentially integrating into routine clinical practice. Although this case report offers clinical insights, further clinical trials are required to validate these findings.

## Data Availability

The original contributions presented in the study are included in the article/supplementary material, further inquiries can be directed to the corresponding authors.
